# Routine ICU admission after brain tumor surgery: retrospective validation and critical appraisal of two prediction scores

**DOI:** 10.1007/s00701-023-05592-9

**Published:** 2023-04-29

**Authors:** Jan-Oliver Neumann, Stephanie Schmidt, Amin Nohman, Martin Jakobs, Andreas Unterberg

**Affiliations:** Department of Neurosurgery, University Hospital Heidelberg, Heidelberg, Germany

**Keywords:** Craniotomy, Adverse effects, Intensive care units, Patient admission, Risk factors

## Abstract

**Background:**

Routine admission to an intensive care unit (ICU) following brain tumor surgery has been a common practice for many years. Although this practice has been challenged by many authors, it has still not changed widely, mainly due to the lack of reliable data for preoperative risk assessment. Motivated by this dilemma, risk prediction scores for postoperative complications following brain tumor surgery have been developed recently. In order to improve the ICU admission policy at our institution, we assessed the applicability, performance, and safety of the two most appropriate risk prediction scores.

**Methods:**

One thousand consecutive adult patients undergoing elective brain tumor resection within 19 months were included. Patients with craniotomy for other causes, i.e., cerebral aneurysms and microvascular decompression, were excluded. The decision for postoperative ICU-surveillance was made by joint judgment of the operating surgeon and the anesthesiologist. All data and features relevant to the scores were extracted from clinical records and subsequent ICU or neurosurgical floor documentation was inspected for any postoperative adverse events requiring ICU admission. The *CranioScore* derived by Cinotti et al. (Anesthesiology 129(6):1111–20, 5) and the risk assessment score of Munari et al. (Acta Neurochir (Wien) 164(3):635–641, 15) were calculated and prognostic performance was evaluated by ROC analysis.

**Results:**

In our cohort, both scores showed only a weak prognostic performance: the CranioScore reached a ROC-AUC of 0.65, while Munari et al.’s score achieved a ROC-AUC of 0.67. When applying the recommended decision thresholds for ICU admission, 64% resp. 68% of patients would be classified as in need of ICU surveillance, and the negative predictive value (NPV) would be 91% for both scores. Lowering the thresholds in order to increase patient safety, i.e., 95% NPV, would lead to ICU admission rates of over 85%.

**Conclusion:**

Performance of both scores was limited in our cohort. In practice, neither would achieve a significant reduction in ICU admission rates, whereas the number of patients suffering complications at the neurosurgical ward would increase. In future, better risk assessment measures are needed.

**Supplementary Information:**

The online version contains supplementary material available at 10.1007/s00701-023-05592-9.

## Introduction

Routine postoperative admission to an intensive care unit (ICU) following brain tumor surgery has been a common practice for many years [6–22]. The main rationale behind this approach is the concern that depressed neurologic function can quickly lead to life-threatening conditions. Postoperative complications such as intracerebral hematoma or status epilepticus need to be diagnosed and treated as soon as possible to prevent permanent neurological compromise. Furthermore, as neurosurgical procedures have traditionally been performed in deep general anesthesia and were often time-consuming, early recovery was often difficult to achieve and sometimes required continued ventilation following the procedure. However, there has never been a consensus on the best approach to this problem, and current practice differs greatly between healthcare systems and individual institutions.

Technical advances in neurosurgery and anesthesiology have led to much shorter procedure durations and much faster neurologic recovery in recent decades. Various studies have shown that the overall complication rate is low nowadays, even for the elderly [19]. Consequently, most patients receive “intensive monitoring” rather than “intensive care” when being admitted to the ICU postoperatively.

Some studies have advocated the cost-efficiency and safety of postoperative surveillance at the post-anesthesia care unit (PACU) and/or the neurosurgical ward [3, 9, 22], and small studies have even proposed a protocol for same-day discharge in selected patients [3, 23]. Various risk factors for a higher likelihood of complications have been suggested in non-systematic reviews or smaller studies [2–4, 9, 10, 17]. Among these are comorbidities such as cardiovascular disease, diabetes, higher age, surgery-associated aspects (e.g., duration, blood loss, vasopressor therapy), tumor size, or certain surgical positions. However, the inclusion criteria vary greatly; some studies include patients with infratentorial tumors [2–5, 17, 20], while others include stereotactical biopsies and/or transsphenoidal approaches which carry a different postoperative risk [4, 10, 16, 20, 21]. Sometimes, the protocols for patients not transferred to ICU include hourly neurological exams performed by nurses and continuous or hourly monitoring of vital parameters which might not be possible in every hospital due to a lower nurse-to-patient ratio [4, 13]. Summing up, studies depict the variety of protocols in neurosurgical departments all over the world.

A systematic review in 2018 performed by de Almeida et al. concluded that routine postoperative admission “may not benefit carefully selected patients” but advises caution due to the lack of prospective studies [6].

With an aging population, the overall number of brain tumor operations is rising constantly. In contrast, ICU capacities are limited due to staff shortages and restricted health budgets. The global COVID-19 pandemic has highlighted the competition of various medical disciplines for these limited ICU resources [14, 20, 21]. This especially affected procedures than require or are thought to require postoperative ICU surveillance and need to be performed in a reasonable time frame, such as neurooncological surgery.

While the usefulness of routine ICU admission has been challenged by many authors, the indication for routine admission remains unclear, but this practice still has not changed in many places, mainly due to the lack of reliable data for preoperative risk assessment.

Motivated and in response to the dilemmas laid out above, risk prediction scores for postoperative complications following brain tumor surgery have been derived from prospective cohorts [5, 10, 15, 18] in recent years.

The CranioScore published in 2018 by Cinotti et al. was explicitly designed to predict postoperative complications in brain tumor surgery [5]. Derived by multivariate logistic regression from a learning cohort of 1094 cases, it was validated in an independent, prospective multicenter cohort of 830 patients in six university hospitals in France.

Munari et al. recently published another score to predict the necessity for ICU admission against direct transfer to the neurosurgical floor following initial postoperative surveillance in the PACU for 6–8 h [15].

At our institution, a major academic neurosurgical center in Germany, we have historically been following the traditional approach of admitting roughly 90% of all brain tumor resection cases to the ICU for postoperative surveillance. While we are eager to question this practice, patient safety concerns and potential legal issues need to be addressed, since ICU surveillance has been a de facto standard in Germany for many years [11]. Therefore, before potentially changing our current protocol, we have aimed to scrutinize the available methods to objectively estimate the need for ICU surveillance. As a consequence, we assessed the applicability, performance, and safety of the risk scores of Cinotti [5] and Munari et al. [15] in a consecutive cohort of 1000 adult patients undergoing elective brain tumor resection.

## Materials and methods

### Study design

This is a retrospective, single-center study to validate the prediction scores published by Cinotti and Munari et al. [5, 15] in a large cohort of consecutive patients undergoing elective craniotomy for brain tumor.

### Patient population

One thousand consecutive adult patients undergoing elective brain tumor resection at our institution between January 2019 and July 2020 (19 months) were included in this study. Patients with craniotomy for other causes such as cerebral aneurysms, microvascular decompression, and others were excluded as well as stereotactical biopsies or transsphenoidal approaches.

The decision for postoperative surveillance on the ICU was made by joint judgment of the operating surgeon and the anesthesiologist. In order to maintain proper planning and optimal ICU and operating room (OR) utilization, a first decision is made during the morning call. Any intra- or postoperative adverse events might cause a reversal of a preliminary decision not to admit a patient to ICU. If no ICU bed is available for a patient which is considered by expert opinion to be in need of it, the case is postponed to the following day.

The majority of 917 (92%) patients were scheduled for admission to the ICU, and only 83 (8%) individuals were planned for direct transfer to the neurosurgical ward after short post-anesthesia surveillance in the PACU.

Criteria for ICU surveillance were all patients except those with a small, supratentorial tumor < 2 cm in diameter and young, otherwise healthy patients (< 50 years) without neurological deficit preoperatively. In case of available excess capacity, those patients were transferred to the ICU as well.

### Data collection

All features relevant to the scores were extracted from clinical records and the preoperative magnet resonance (MR) images were reviewed for tumor size and volume. The anesthesia report and subsequent ICU or neurosurgical floor documentation was scrutinized for any intra- or postoperative adverse events as well as physiological parameters relevant to both scores. Table S1 shows a definition of relevant postoperative events.

Seven patients with missing data points were excluded from the analysis. Based on all recorded events and following the same methodology as in the original publications, a determination was made from an *ex-post* perspective, if a patient should have been admitted to the ICU or could have been observed on the neurosurgical ward.

### Investigated prediction scores

The *CranioScore* derived by Cinotti et al. [5] is a logistic regression model derived from a monocentric learning cohort of 1094 cases to predict any of these conditions post brain surgery:

- moderate to severe intracerebral bleeding (confirmed on CT), intracranial hypertension (confirmed on CT or measured by probe/EVD above 20 mmHg), status epilepticus or seizures, need for tracheal intubation and mechanical ventilation after the neurosurgical procedure, impaired consciousness (GCS <  = 13), unmanageable agitation, severe swallowing disorders leading to aspiration and respiratory failure, unexpected severe motor deficit (motor score at or above 3), and finally death in the perioperative period.

The log-odds are calculated as

$${L}_{CS}= -4.8094+1.5149G+1.0534B+0.00878S+0.5114M+0.5164T +0.0118RRmax-0.0130RRmin+0.2981D$$

with following independent variables: *preoperative GCS* ≤ *14 (G), history of prior brain surgery (B), largest tumor diameter on MRI (S), midline shift* > *3 mm on MRI (M), transfusion of erythrocytes or platelets (T), maximum and minimum blood pressure during surgery (RRmax/RRmin), procedure duration in minutes from incision to closure (D)*.

Munari et al. [15] followed a more limited approach by excluding any posterior fossa tumors and/or patients with conscious impairment prior to surgery. Furthermore, patients had to remain under surveillance in the PACU for 6–8 h prior to transfer to the ICU or the neurosurgical ward. The aim of the score was to predict any complication or condition during PACU observation that ultimately led to ICU admission by the treating physician. Logistic regression from a learning cohort of 287 patients revealed surgery length, tumor value, and American Society of Anesthesiologists Physical Status (ASA-PS) as significant factors for ICU admission.

A simple scoring system of + 2 points for every hour of surgery, + 0.5 for every 5 ${cm}^{3}$ of tumor volume, and + 4, + 8, or + 12 points for ASA-PS scores of 2, 3, or 4 were proposed and validated in a cohort of 133 patients. A threshold of 12.5 points was recommended for ICU admission.

### Statistics

The sample size of 1000 patients was chosen based on an estimated minimum of 10% postoperative adverse events.

Descriptive statistics were calculated in Python (3.8) using the SciPy (version 32) and pandas (version 23.32) packages. The discriminative performance of the score was assessed by calculating the area under the curve of the receiver operating characteristic (AUC-ROC). Confidence intervals were calculated using a t-distribution with an $\alpha <5\%$.

### Ethics approval

The study was performed in accordance with the declaration of Helsinki. The design of this study and the retrospective collection and analysis of patient data were approved by the institutional review board of the University of Heidelberg (S-275/2021). The requirement of informed consent for collection and processing of anonymized patient data was waived.

## Results

### Demographics and comorbidities

The full cohort consisted of 552 female (55%) and 448 male (45%) patients between 18 and 88 (mean 57 ± 15) years. The vast majority (94%) were preoperatively assigned to ASA-PS classes II and III. The most common comorbidities were arterial hypertension (41%) and diabetes (11%). Twenty-six percent of patients had a history of prior craniotomy and 23% a history of seizures. The burden of preoperative neurological deficit was low with a mean modified National Institute of Health Stroke Scale (mNIHSS) of 1 (range 0–11) and a median Glasgow Coma Scale (GCS) of 15 (range 11–15). Because the score of Munari et al. [15] excluded posterior fossa tumors, a corresponding subgroup of 777 patients was used to assess this score. There were no noteworthy differences concerning demographics and comorbidities between these subgroups. Table 1 summarizes the demographics and comorbidities of both patient cohorts.

**Table 1:** Demographics and comorbidities

Parameter	Supra- and infratentorial cases (*n* = 1000)	Supratentorial cases only (*n* = 777)
Age (years)		
Range	18–88	18–88
Mean ± SD	57 ± 15	58 ± 15
Median (IQR)	58 (47–68)	58 (47–68)
Gender		
Female	552 (55%)	422 (54%)
Male	448 (45%)	355 (46%)
Body mass index		
Range	13–46	13–46
Mean ± SD	26 ± 5.0	27 ± 5.0
Median (IQR)	26 (24–29)	26 (23–29)
ASA class		
I	56 (6%)	43 (6%)
II	577 (58%)	432 (56%)
III	358 (36%)	295 (38%)
IV	9 (1%)	7 (1%)
V	0 (0%)	0 (0%)
mNIHSS		
Range	0–11	0–11
Mean ± SD	1 ± 1,5	1 ± 1,5
Median (IQR)	0 (0–1)	0 (0–2)
Preoperative GCS		
Range	11–15	11–15
Mean ± SD	15 ± 0.3	15 ± 0.3
Median (IQR)	15 (15–15)	15 (15–15)
Arterial hypertension	412 (41%)	321 (41%)
Diabetes mellitus	113 (11%)	94 (12%)
Anticoagulation / antiplatelet medication	136 (14%)	108 (14%)
History of craniotomy	234 (23%)	205 (26%)
History of epilepsy	259 (26%)	250 (32%)
History of clotting disorders	19 (2%)	13 (2%)
History of thromboembolism	40 (4%)	32 (4%)

### Intracranial pathologies, radiological features, and procedures

Craniotomy for removal of meningioma (36%), cerebral metastases (19%), or glioblastoma (16%) comprised almost three quarters of all procedures. Tumor location was supratentorial in 78% of cases, while 22% of the tumors were located infratentorial. Intraventricular tumors accounted for 25 (3%) of cases.

The mean tumor volume on preoperative MRI was 22 ± 31.4 (SD) ml. A midline shift of 3 mm or greater was seen in 19% of cases. Positioning was supine in 67%, prone in 13%, and lateral in 7% of cases. The semi-sitting position was chosen in 13% of the procedures. Mean duration from incision to closure was 236 ± 102 (SD) minutes.

Table 2 contains more detailed information about pathologies, radiological features, and procedure characteristics in both cohorts. The subgroup of supratentorial cases showed minor differences in tumor histology, duration of procedure, and positioning compared to the cohort off all cases.

**Table 2:** Histology, radiology, and procedure data

Parameter	Supra- and infratentorial cases (*n* = 1000)	Supratentorial cases only (*n* = 777)
Suspected histology of tumor		
Meningoma	364 (36%)	297 (38%)
Metastasis	188 (19%)	141 (18%)
Glioblastoma	164 (16%)	164 (21%)
Astrocytoma °III	39 (4%)	38 (5%)
Astrocytoma °II	74 (7%)	72 (9%)
Others	171 (17%)	65 (8%)
Tumor location		
Frontal	371 (37%)	371 (48%)
Temporal	203 (20%)	203 (26%)
Parietal	109 (11%)	109 (14%)
Occipital	69 (7%)	69 (9%)
Brainstem	129 (13%)	-
Cerebellar	94 (9%)	-
Intraventricular	25 (3%)	25 (3%)
Tumor volume (ml)		
Range	1–272	1
Mean ± SD	22 ± 31.4	34.0
Median (IQR)	10 (3–27))	12 (4–32)
Greater size (mm)		
Range	2–124	2–124
Mean ± SD	35 ± 17.1	37 ± 17.9
Median (IQR)	32 (22–45)	35 (23–47)
Midline shift > = 3 mm	186 (19%)	181 (23%)
Duration of procedure (minutes)		
Range	20–740	53–623
Mean ± SD	236 ± 102.2	229 ± 98.8
Median (IQR)	218 (162–288)	215 (162–289)
Positioning		
Supine	673 (67%)	666 (85%)
Prone	130 (13%)	84 (11%)
Lateral	71 (7%)	22 (3%)
Sitting	125 (13%)	5 (1%)
SBP maximum intraoperative (mmHg)		
Range	90–180	90–180
Mean ± SD	135 ± 16	135 ± 16
Median (IQR)	130 (120–145)	130 (120–145)
SBP minimum intraoperative (mmHg)		
Range	50–140	50–140
Mean ± SD	100 ± 9.9	100 ± 9.8
Median (IQR)	100 (90–100)	100 (90–100)
Transfusion of erythrocytes, plasma, or platelets	20 (2%)	13 (2%)

### Postoperative ICU events

During postoperative surveillance on the ICU (92%, *n* = 917) or PACU/neurosurgical floor (8%, *n* = 83), adverse events requiring ICU intervention occurred in 158 cases (16%). The total number of events was 284 in all cases. In the subgroup of supratentorial cases, these figures were similar with 208 events in 121 cases (16%). Table 3 summarizes the frequencies of the ICU events encountered in the postoperative period for both groups. Two postoperative events occurred after the patient had already been transferred from the PACU to the neurosurgical floor: the first patient showed arterial hypotension requiring vasopressor therapy, and the second patient deteriorated neurologically with hemiparesis and reduced vigilance. A computer tomography (CT) scan revealed an intracerebral hematoma requiring operative revision.

**Table 3:** Postoperative ICU events

Postoperative ICU events	Supra- and infratentorial cases (*n* = 1000)	Supratentorial cases only (*n* = 777)
Cases with at least one event	158 (15.8%)	121 (15.8%)
Total number of events	284	208
CPR	4 (0.4%)	4 (0.5%)
Reintubation	12 (1.2%)	8 (1.0%)
Return to OR	13 (1.3%)	10 (1.3%)
Mechanical ventilation	25 (2.5%)	12 (1.5%)
Vasopressors	22 (2.2%)	16 (2.1%)
Impaired consciousness (GCS ≤ 13)	34 (3.4%)	21 (2.7%)
Intracranial hypertension treated by		
CSF drainage	22 (2.2%)	10 (1.3%)
Mannitol	47 (4.7%)	38 (4.9%)
Seizure	42 (4.2%)	41 (5.3%)
Hemiparesis (grade ≤ 3/5)	46 (4.6%)	40 (5.1%)
Swallowing disorder	17 (1.7%)	8 (1.0%)
Death in the perioperative period	0 (0%)	0 (0%)

**Fig. 1:**
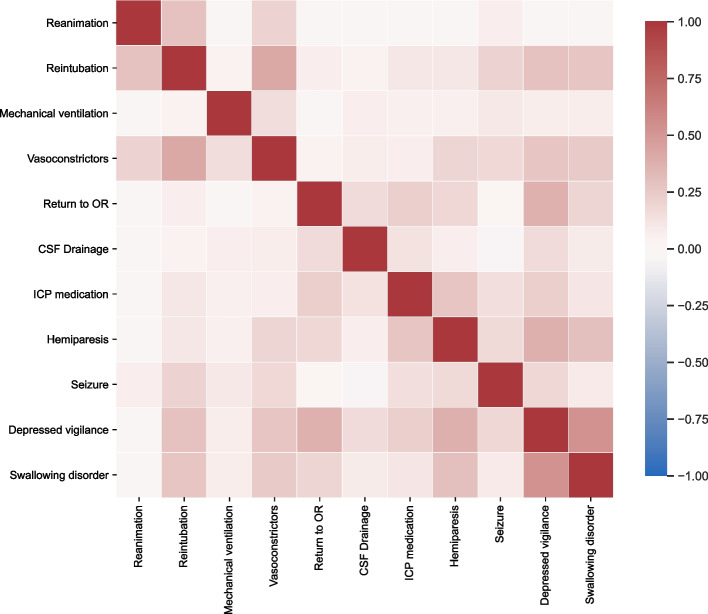
Correlation heatmap of 284 postoperative ICU events in 1000 patients

When looking for coincidences between postoperative ICU events, the heatmap (cf. Figure 1) reveals a clustering of general intensive care (reanimation, reintubation, ventilation, and vasoconstrictors) and neurological events like hemiparesis, seizures, and others. Furthermore, there is a linkage between severe neurologic symptoms like depressed vigilance, swallowing disorders, and generalized seizures and subsequent events like reintubation, ventilation, and necessity of vasoconstrictor therapy.

The heatmap (Cohen’s kappa) shows a clustering of predominantly anesthesiological (reanimation, reintubation, ventilation, and vasoconstrictors) and neurological events like hemiparesis, seizures, and others. Between these distinct groups, depressed vigilance, swallowing disorders, and seizures were the most common triggers for the first group of events.

### Prognostic performance of prediction scores

**Fig. 2:**
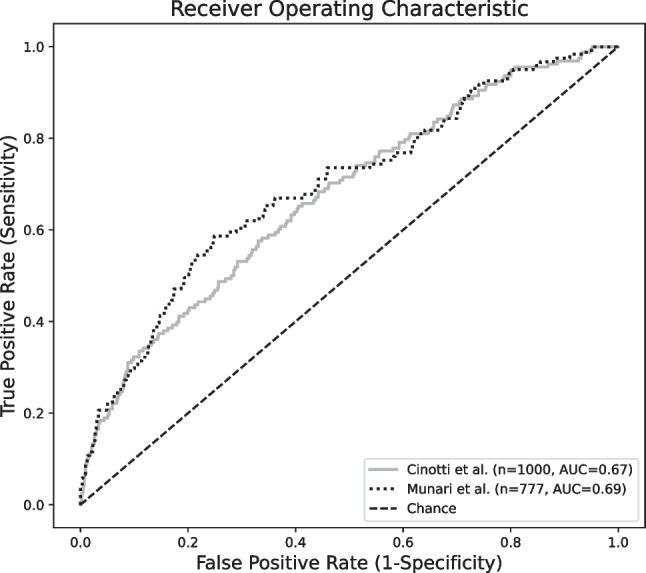
Receiver operating characteristics

The CranioScore [5] achieved an area under the curve of the receiver operating characteristic (AUC-ROC) of 0.67 when used to predict the occurrence of postoperative events in the patient cohort with supra- and infratentorial tumors (*n* = 1000). The prediction instrument of Munari et al. [15] yielded an AUC-ROC of 0.69 in the subgroup of supratentorial tumor cases (*n* = 777). The ROC curves for both scores are shown in Fig. 2.

Applying the recommended threshold of 3% to predict the need of postoperative ICU surveillance, the CranioScore yields a negative predictive value (NPV) of 91%, a sensitivity of 82%, and a specificity of 35% with an ICU admission rate of 64%. The NPV can be raised above the 95% limit with a lower threshold of 2%, but specificity will drop to 17% with a subsequent ICU admission rate of more than 85%.

Munari’s score shows a very similar performance at the recommended 12.5-point threshold with a NPV of 91%, sensitivity of 82%, specificity of 35%, and ICU admission rate of 68%. Lowering the threshold to 10 points to achieve 95% NPV would lead to 84% ICU referrals.

**Fig. 3:**
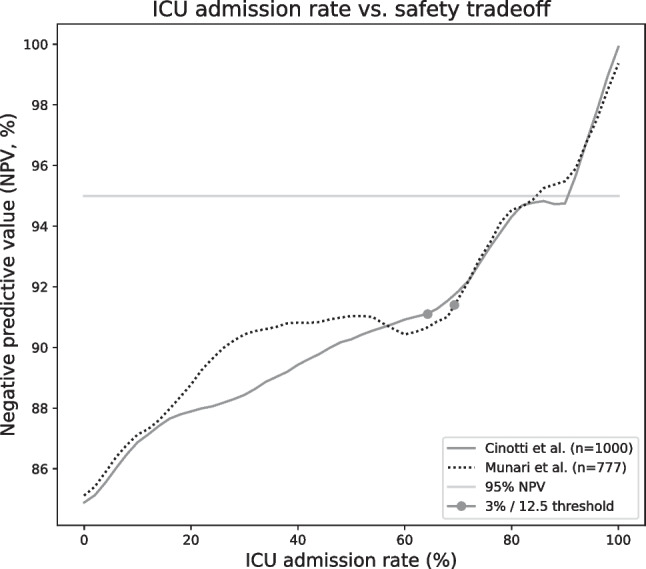
ICU admission rate vs. safety tradeoff

Figure 3 demonstrates the possible tradeoffs between NPV and admission rates for both scores.

The CranioScore [5] (grey line) achieved an area under the curve of the receiver operating characteristic (AUC-ROC) of 0.67 when used to predict the occurrence of postoperative events in the patient cohort with supra- and infratentorial tumors (*n* = 1000). The prediction instrument of Munari et al. [15] (dotted line) yielded an AUC-ROC of 0.69 in the subgroup of supratentorial tumor cases (*n* = 777).

A tradeoff between ICU admission rate and safety is required when applying both scores in a clinical setting. At the recommended CranioScore [5] threshold of 3%, it is necessary to admit 64% of all cases to the ICU to achieve a negative predictive value (NPV) of roughly 91%. Munari’s [15] score shows a very similar performance at the recommended 12.5-point threshold. When aiming at a desired NPV of more than 95%, the ICU admission rate is raised beyond 80% for both scores.

## Discussion

In this study, we examined the prognostic performance of two prediction scores for ICU surveillance after brain tumor resection.

The current management in our cohort can be regarded as very conservative with an ICU admission rate of 92% with an excellent negative predictive value of 96%. Our requirement for any risk prediction algorithm is that it should lower ICU admission rate while not compromising patient safety compared to our current practice. Therefore, a negative predictive value of 95% represents the desired safety level. This figure is in-line with the 5% chance for type II errors (significance level) that is commonly accepted in medical science.

The CranioScore achieved an AUC-ROC of 0.67 in our cohort of 1000 patients undergoing elective craniotomy for brain tumor. This figure is at the lower limit of the 95% CI of [0.64; 0.76] reported in the validation cohort of the original publication [5]. AUC-ROC values below 0.7 have to be regarded as weak prognostic performance [12].

In our setting, to maintain adequate patient safety as laid out before, the decision threshold would have to be lowered down to 2% to raise the NPV above the 95% level. As this would lead to ICU admission rates above 85%, adopting the CranioScore would not lead to a meaningful improvement of our admission strategy.

Furthermore, four of the eight independent variables of the score (duration, maximum and minimum blood pressure, and the need for transfusions) will be available only after the procedure has finished. Consequently, the CranioScore cannot be used to support procedure scheduling decisions.

In summary, although derived using logistic regression from the largest cohort so far (*n* = 1094) and prospectively validated in a second, multicentric cohort (*n* = 830), we consider the CranioScore not to be useful in our setting.

Munari’s score was designed to decide if a patient can be safely transferred to the neurosurgical ward following extubation in the operating room and subsequent 6–8-h surveillance in the PACU [15]. This approach effectively uses the PACU as a “fast-track” ICU, which may not be feasible in many institutions. This is a relevant limitation of their scoring system. Furthermore, it was based on very small training (*n* = 287) and validation cohorts (*n* = 133) and is limited to supratentorial tumors.

The original study reported a 95% CI of [0.668; 0.880] for ROC-AUC. The broad confidence interval is a direct result of the small number of patients in the training and validation cohorts. When assessing the performance in our setting in a much larger sample (supratentorial tumors only, *n* = 777), an AUC-ROC of 0.69 was found. This result is only slightly better than the performance of the CranioScore and, again, too low to be helpful to maintain patient safety while significantly reducing ICU admission rates.

We considered two more available predictions scores as candidates for validation in this study, but after carefully evaluating their derivation methods and intended use cases, excluded them from our analysis.

Franko et al. proposed a simple score of three items (Karnofsky performance status < 70 points, general anesthesia and early postoperative complications) to predict the risk of ICU interventions [8]. The score is limited to supratentorial tumors and considers early complications within 4 h of surgery as independent variable, effectively relocating postoperative surveillance from the ICU to the PACU. Furthermore, training (*n* = 200) and validation cohorts (*n* = 100) were too small when taking the low incidence of postoperative complications into account. Finally, the training cohort included 37% awake craniotomies, which represents a preselection of “low-risk” cases in the training cohort and might lead to underestimation of postoperative complications.

Rozeboom et al. recently published a multivariable prediction model for postoperative intensive care unit stay in a broad surgical population [18]. Joining data from the National Surgical Quality Improvement Program (NSQIP) of the American College of Surgeons with in-hospital and ICU data from five surgical centers, a large dataset of 34,568 patients from eight surgical specialties was synthesized. It included 2616 neurosurgical cases of all kinds including spinal cases. The overall prognostic performance for ICU admission in this broad population was very high with an overall ROC-AUC over 90%, but the sample included outpatient and emergency surgery cases, and ICU admission rates were highly correlated with the surgical specialty. Furthermore, the authors admitted that ICU referral in the neurosurgical subgroup (72% no ICU use, 28% ICU use) was based mostly on surgeon’s request rather than an objective need for it. It seems evident that the remaining prognostic variables (ASA-PS, age, functional status, relative procedure complexity) would not yield a reliable prediction in our intended use case.

At this point, a general limitation of all ICU prediction instruments has to be addressed: postoperative care protocols as well as ICU, PACU, and surgical ward equipment and staffing differ considerably between countries individual hospitals [1]. There is no consensus and/or rigorous definition what types of events are relevant for a patient’s outcome and how they should be handled. Consequently, the reported rates of postoperative events requiring an ICU after neurosurgical procedures vary widely in the literature.

Hanak et al. reported an incidence of up to 35% ICU-worthy interventions in 400 neurosurgical tumor cases [10], but most of them were limited to IV blood pressure medication, which could be handled in an non-ICU setting in many cases. Rozeboom reported 28% ICU referrals in their large and diverse cohort, but these were based on surgeons’ request than based on physiological data (see above) [18].

Cinotti’s definition of adverse events, which was used in this study, is more rigorous and led to an incidence of postoperative events of 12% in their cohort and 16% in our study. Other authors have reported lower event rates of 5–9% [15, 17, 20], but in many cases, the exact definition of “relevant” events remained ambiguous.

Although it must be admitted that adverse events occurring on the neurosurgical ward might not necessarily lead to worse outcomes than on the ICU, we argue that the events defined by Cinotti et al. truly represent a relevant threat to the patients’ safety and represent a lowest common denominator for a “high-risk” classification many neurosurgeons and neurointensivists would agree upon.

Our study itself has its own limitations: it is retrospective in nature, and data had to be extracted from clinical records that were not intended for study use and sometimes subject to individual interpretation. We have tried to err on the side of caution in our assessment of postoperative events which is reflected in our comparably high number of 16% postoperative adverse events.

## Conclusion

Although we have determined that the currently available scoring systems are not suitable in our setting, we are convinced that risk assessment and ICU admission policy following brain tumor surgery should be based on objective, evidence-based measures rather than personal preferences and requests.

We hypothesize that the methods used in the studies so far, i.e., logistic regression from a small number of independent variables, are insufficient to attain satisfactory diagnostic power in this complex problem. Other options, which can be implemented in the clinical workflow easily, should be evaluated, i.e., machine learning.

## Supplementary Information

Below is the link to the electronic supplementary material.

Supplementary file1 (PDF 47 KB)

## Abbreviations

ASA-PS: American Society of Anesthesiologists physical status classification system
AUC-ROC: Area under the curve of the receiver operating characteristic
CI: Confidence interval
CPR: Cardiopulmonary resuscitation
CSF: Cerebrospinal fluid
CT: Computer tomography
EVD: External ventricular drainage
GCS: Glasgow Coma Scale
ICP: Intracranial pressure
ICU: Intensive care unit
IQR: Interquartile range
MRI: Magnet resonance imaging
mNIHSS: Modified National Institutes of Health Stroke Scale
NSQIP: National Surgical Quality Improvement Program
NPV: Negative predictive value
OR: Operating room
PACU: Post-anesthesia care unit
RR: Blood pressure
SBP: Systolic blood pressure
SD: Standard deviation
