# Supplementary material for: Routine ICU admission after brain tumor surgery: retrospective validation and critical appraisal of two prediction scores
Source: Acta Neurochir (Wien). 2023 Apr 29;165(6):1655–64. doi: 10.1007/s00701-023-05592-9 (PMC10147995; doi:10.1007/s00701-023-05592-9)
Supplement: Supplementary file 1 — Supplementary file1 (PDF 47 KB) [file 701_2023_5592_MOESM1_ESM.pdf]

Table S1 – Definition of postoperative ICU events

**Postoperative ICU events**	
Reintubation	Reintubation for any reason other than revision surgery
Return to OR	Any surgery due to complications within 24 hours
Mechanical ventilation	Any postoperative ventilation > 4 hours
Vasopressors	Application of over 0,4 mg norepinephrine per hour to hold MAP > 60 mmHg
Impaired consciousness	GCS <= 13
Intracranial hypertension	Mannitol therapy or CSF drainage
Seizure	Generalized seizure or recurring focal seizure
Hemiparesis	Grade <= 3/5
Swallowing Disorder	Impaired swallowing requiring a gastral tube or parenteral nutrition
CPR	
Death in the perioperative period	

Routine ICU Admission After Brain Tumor Surgery: Retrospective Validation and Critical Appraisal of Two Prediction Scores

Jan-Oliver Neumann[1§], Stephanie Schmidt[1§], Amin Nohman[1], Martin Jakobs[1] and Andreas Unterberg[1]

[1]Department of Neurosurgery, University Hospital Heidelberg, Germany

[§]Both authors contributed equally

Corresponding author: PD Dr. Jan-Oliver Neumann
Department of Neurosurgery
University Hospital Heidelberg
Im Neuenheimer Feld 400
D-69120 Heidelberg
+49-6221-56-38096
Jan-Oliver.Neumann@med.uni-heidelberg.de
